# Supplementary material for: Cell-Type-Specific Proteogenomic Signal Diffusion for Integrating Multi-Omics Data Predicts Novel Schizophrenia Risk Genes
Source: Patterns (N Y). 2020 Sep 2;1(6):100091. doi: 10.1016/j.patter.2020.100091 (PMC7518509; doi:10.1016/j.patter.2020.100091)
Supplement: Document S1. Figure S1 [file mmc1.pdf]

PATTER, Volume *1*

Supplemental Information

Cell-Type-Specific Proteogenomic Signal Diffusion for Integrating Multi-Omics Data Predicts Novel Schizophrenia Risk Genes

Abolfazl Doostparast Torshizi, Jubao Duan, and Kai Wang

Supplementary Figures

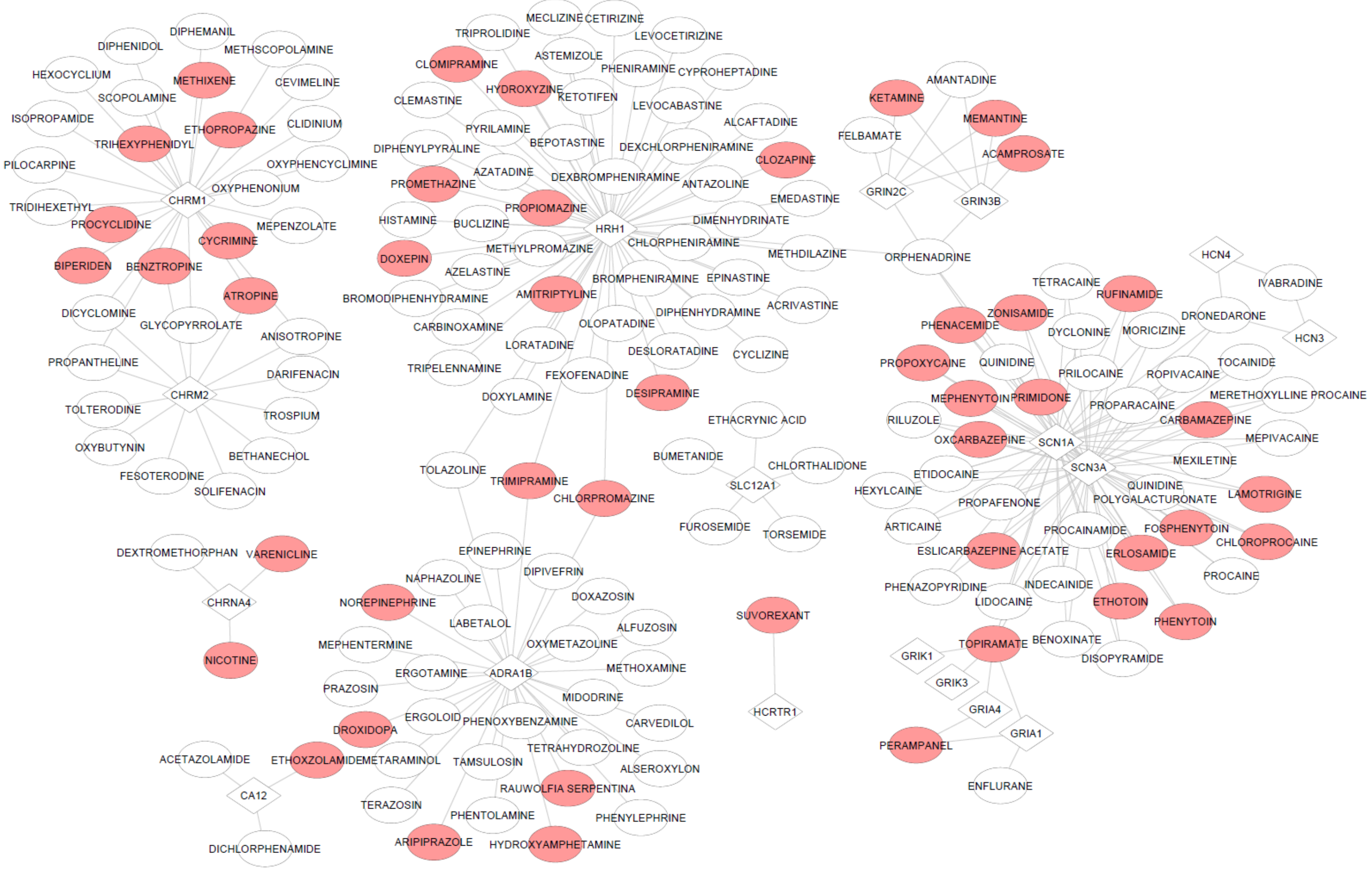

Supplementary Fig 1. Drug-target network representation of MAPSD risk genes and their corresponding FDA-approved drugs. Genes are denoted by diamonds and drugs are denoted by ovals. Red nodes represent the drugs developed for the diseases related to central nervous system.
