# Supplementary material for: Cell-Type-Specific Proteogenomic Signal Diffusion for Integrating Multi-Omics Data Predicts Novel Schizophrenia Risk Genes
Source: Patterns (N Y). 2020 Sep 2;1(6):100091. doi: 10.1016/j.patter.2020.100091 (PMC7518509; doi:10.1016/j.patter.2020.100091)
Supplement: Document S2. Article plus Supplemental Information [file mmc5.pdf]

Patterns

Article

Cell-Type-Specific Proteogenomic Signal Diffusion for Integrating Multi-Omics Data Predicts Novel Schizophrenia Risk Genes

Graphical Abstract

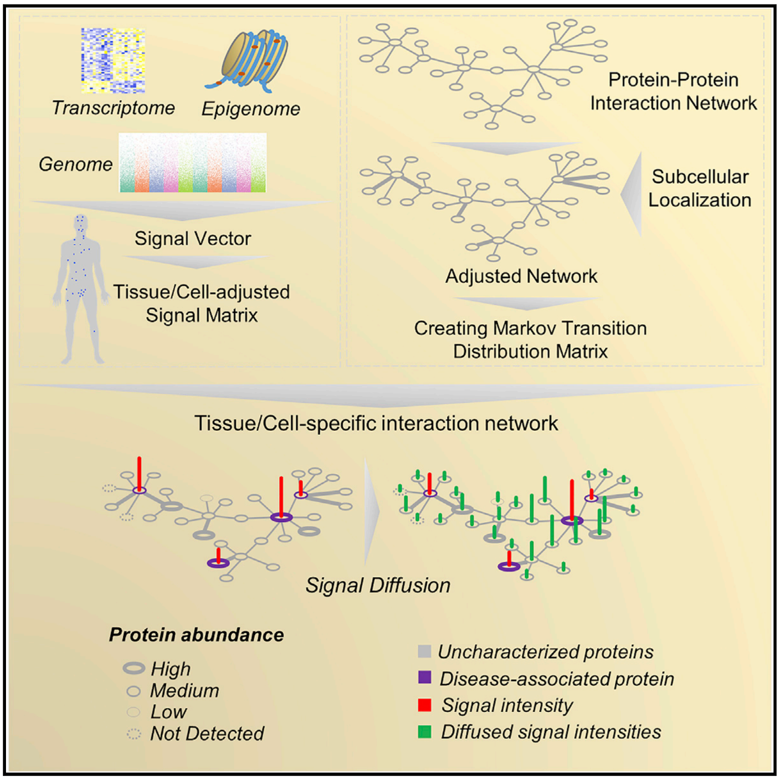

Highlights

- MAPSD models protein trafficking for disease modeling.
- Integrated proteome-genome modeling identifies novel schizophrenia risk genes.
- Schizophrenia risk genes are involved in different stages of neurodevelopment.
- Schizophrenia risk genes may converge in modules in interaction networks.

Authors

Abolfazl Doostparast Torshizi, Jubao Duan, Kai Wang

Correspondence

wangk@email.chop.edu

In Brief

Proteome studies are lagging behind the research on nucleic acids in neuropsychiatric disorders. We present a novel data integration method (called MAPSD) to model protein trafficking maps within the cellular micro-domains in conjunction with other available biological data such as gene expression data to identify novel schizophrenia risk genes. We showed that targeted modeling of disease signatures in sub-regions of each tissue enables us to discover critical therapeutic insights that may not be revealed by the existing approaches.

Doostparast Torshizi et al., 2020, Patterns *1*, 100091
October 9, 2020 © 2020 The Author(s).
https://doi.org/10.1016/j.patter.2020.100091

CellPress

Patterns

CellPress
OPEN ACCESS

Article

Cell-Type-Specific Proteogenomic Signal Diffusion for Integrating Multi-Omics Data Predicts Novel Schizophrenia Risk Genes

Abolfazl Doostparast Torshizi,[1] Jubao Duan,[2,3] and Kai Wang[1,4,5,*]
[1]Raymond G. Perelman Center for Cellular and Molecular Therapeutics, Children's Hospital of Philadelphia, Philadelphia, PA 19104, USA
[2]Center for Psychiatric Genetics, North Shore University Health System, Evanston, IL 60201, USA
[3]Department of Psychiatry and Behavioral Neurosciences, University of Chicago, Chicago, IL 60637, USA
[4]Department of Pathology and Laboratory Medicine, Perelman School of Medicine, University of Pennsylvania, Philadelphia, PA 19104, USA
[5]Lead Contact
*Correspondence: wangk@email.chop.edu
https://doi.org/10.1016/j.patter.2020.100091

THE BIGGER PICTURE Proteins constitute the functional machinery in a cell. Genetic aberrations may cause disrupting the normal functionality of the proteins. On the other hand, biophysical and biochemical properties of proteins vary in distinct tissues mandating separate modeling of proteomic features given the tissue being studied, e.g. brain in case of schizophrenia. Using the concept of signal diffusion in graph theory, we proposed a model, termed MAPSD, which enables us to leverage proteomic properties of different tissues at single cell resolution along with genomic and epigenomic features of a disease in order to predict potential risk genes which cannot be annotated using common univariate approaches. Taking this approach helps create novel therapeutic hypotheses for precision medicine so that more effective treatments with less side effects on other organs can be developed. Application of MAPSD is not restricted to schizophrenia and most of complex diseases can benefit from the method.

1 2 3 4 5 **Proof-of-Concept:** Data science output has been formulated, implemented, and tested for one domain/problem

SUMMARY

Accumulation of diverse types of omics data on schizophrenia (SCZ) requires a systems approach to model the interplay between genome, transcriptome, and proteome. We introduce Markov affinity-based proteogenomic signal diffusion (MAPSD), a method to model intra-cellular protein trafficking paradigms and tissue-wise single-cell protein abundances. MAPSD integrates multi-omics data to amplify the signals at SCZ risk loci with small effect sizes, and reveal convergent disease-associated gene modules in the brain. We predicted a set of high-confidence SCZ risk loci followed by characterizing the subcellular localization of proteins encoded by candidate SCZ risk genes, and illustrated that most are enriched in neuronal cells in the cerebral cortex as well as Purkinje cells in the cerebellum. We demonstrated how the identified genes may be involved in neurodevelopment, how they may alter SCZ-related biological pathways, and how they facilitate drug repurposing. MAPSD is applicable in other polygenic diseases and can facilitate our understanding of disease mechanisms.

INTRODUCTION

The emergence of omics technologies has revolutionized neuropsychiatric research[1] by generating high-throughput genomic data, bridging genome and transcriptome to phenome.[2] For example, genome-wide association studies (GWAS), such as the Psychiatric Genomics Consortium (PGC)[3] and the CLOZUK consortium[4] have created a repertoire of tens of thousands of samples worldwide, leading to the discovery of many common variants associated with schizophrenia (SCZ). While such studies mark important milestones in SCZ research, they face critical challenges with regard to extracting novel biological

Check for updates

This is an open access article under the CC BY license (http://creativecommons.org/licenses/by/4.0/).

insights and finding additional therapeutic targets or pathways. In fact, only one recognized drug target dopamine receptor D2 (*DRD2*) for SCZ has been re-identified by GWAS.[5] It is not trivial to accurately pinpoint the corresponding risk genes in each GWAS risk locus, as such loci may cover a myriad of genes while the genuine causal variants may be away from the top-ranking single nucleotide polymorphisms (SNPs).[6]

In addition to genetic association studies, tremendous efforts have been made over the years to understand the machinery of gene regulation. Whole-body proteomics data, such as the Human Protein Atlas,[7,8] now delineates protein expression not only across tens of various tissues but at certain cell types, while drawing their subcellular localization. Moreover, large-scale epigenomics data, such as Functional Annotation of the Mammalian Genome 5[9] and genome-scale chromosome conformation capture[10,11] technology have brought about unprecedented opportunities to elucidate long-range interactions among genetic loci. Given that individual omics data serve as complementary elements to each other, integrating multi-omics data types can strengthen subtle disease signals from risk genes.[5,12,13] In fact, such multi-omics perspective amplifies signals from genetic loci with small effect sizes, and help support converging evidence on certain biological processes. This is of critical importance in understanding polygenic diseases, such as SCZ.

The current available omics data on SCZ are predominantly related to those of nucleic acids, e.g., genomics, transcriptomics, and epigenomics, while the use of proteomics information is quite limited.[14] As the functional machinery in a cell, proteins essentially reflect the functional consequences of genome, epigenome, and transcriptome. Although proteins are treated as proxies of gene functions, multiple lines of evidence report a maximum of 60% correlation between the gene and protein expression levels in certain organisms.[15,16] Moreover, functionality of proteins is not restricted to their abundances, where other determinants such as biochemical and physical properties, such as subcellular localization, protein-protein interactions (PPIs), and post-translational modifications affect such functions.[17] This mandates an inclusive in-depth analysis of the proteome and its physical and biochemical properties, not only at the tissue level but at the cell resolution in SCZ. Although proteomic investigations have been historically hampered due to the lack of low-cost and reliable high-throughput assay platforms,[18,19] there have been recent advances in improving the mass spectrometry-based proteomics platforms,[20,21] which has resulted in the generation of valuable resources, such as the Human Protein Atlas.[7,8] On the other hand, subcellular fraction allows probing enrichment of proteins in micro-domains within cells (such as neurons), and offers insights into understanding the intra-cellular trafficking trajectories of proteins. There have been several proteomic studies on SCZ,[22–25] which mainly focus on observing the differential expression of proteins in postmortem brains, without taking into account tissue- or cell-specific biochemical and biophysical interactions. For a full review on proteome studies in SCZ, refer to Borgmann-Winter et al.[14]

In this study, we introduce MAPSD (Markov affinity-based proteogenomic signal diffusion), a multi-omics network-based computational method to identify novel risk genes for polygenic diseases. MAPSD leverages multiple layers of omics information, as well as the under-studied proteome subcellular localization patterns and tissue-wise cell-specific abundances of proteins in tens of different tissues and a wide range of cells, followed by propagating the biological signals across the human interactome to characterize potential disease-associated risk genes. The proposed model has several unique advantages, including (1) it uses protein trafficking information in subcellular micro-domains in 131 tissues and cell types, including multiple regions in the brain from the Human Protein Atlas;[7,8] (2) MAPSD uses five layers of omics data including differentially expressed (DE) genes,[2] GWAS hits,[3,4] rare and *de novo* mutations,[26] differentially methylated genes,[27–29] and chromatin accessibility data;[30] and (3) MAPSD can effectively model interactions of genome, epigenome, transcriptome, and proteome at a single-cell resolution. Although we used SCZ as a test case in the study, MAPSD is flexible and can be effectively applied to other polygenic diseases other than SCZ. The outcome of MAPSD is accurate prediction of risk levels of all human genes in SCZ, which has led to the identification of a set of new candidate genes for SCZ. Our functional evaluation on these candidate genes indicate how the MAPSD-identified genes are predominantly enriched in certain cell types within specific brain regions. In particular, the novel candidate genes identified by us are enriched for the targets of approved drugs for brain disorders and suggest opportunities for repurposing existing therapies for SCZ.

RESULTS

Overview of the MAPSD Framework

MAPSD is a multi-step tissue/cell-specific proteogenomic method to identify risk genes through leveraging complementary biological signals from distinct omics data modalities. The overall structure of MAPSD is provided in Figure 1. MAPSD starts with a large-scale PPI network which is assembled from multiple sources[31–34] (see Experimental Procedures). Using the PPI network, an affinity matrix is created. This matrix is binary in which if two nodes (proteins) interact then their corresponding matrix elements will be 1, otherwise 0. The PPI network is then adjusted to include molecular trafficking patterns. This adjustment is conducted using the subcellular localization data from the Human Protein Atlas (Figure 2A). The rationale behind this adjustment is that if two proteins being connected in the PPI network co-localize in the same micro-domain within the cell, then they are more likely to be interacting with each other. In total, 32 micro-domains have been used in this study. Therefore, the weight of connecting edges of co-localized proteins in the PPI network is amplified by a factor of 1.5, while the remaining edges have a weight of 1 (see Experimental Procedures). Using the adjusted affinity matrix, the Markov transition distribution matrix M is created. Using graph Laplacian concept in graph theory, a one-step probability distribution from each node to its neighbors is computed (see Experimental Procedures).

The multi-omics datasets have been collected from multiple sources (see Experimental Procedures). We used SCZ as a test case in our study to evaluate the MAPSD approach, due to the availability of large-scale genomics, transcriptomics, and epigenomics datasets on SCZ. Five layers of omics data have been used in this study, including DE genes,[2] GWAS hits,[3,4] rare and *de novo* mutation loci,[26] differentially methylated loci,[27–29] and loci being differentially accessible in open

CellPress
OPEN ACCESS

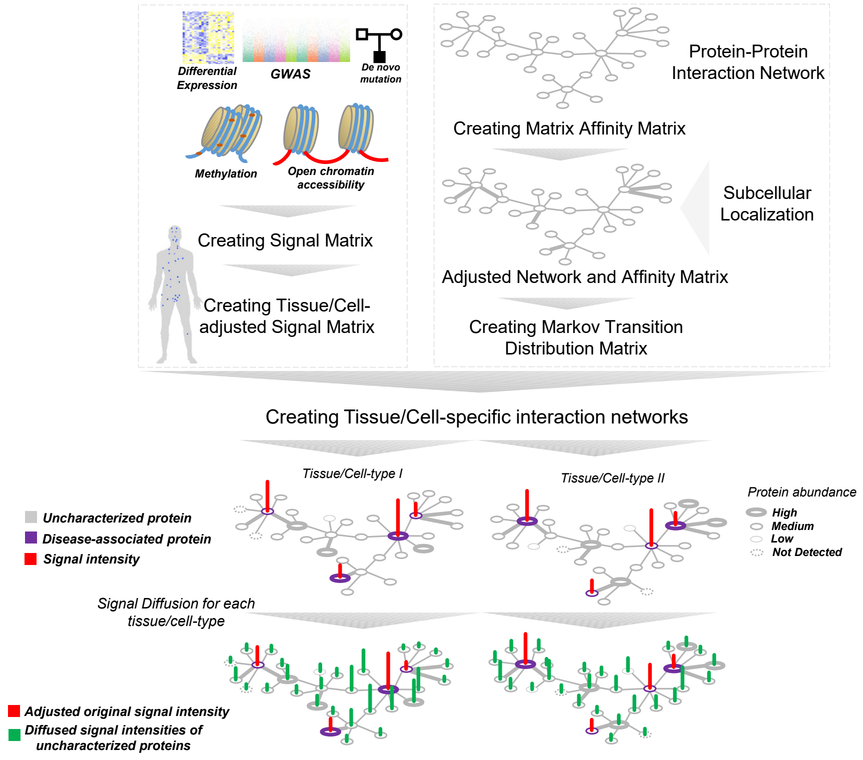

Figure 1. The Structure of MAPSD

MAPSD steps include: creating the protein-protein interaction network followed by adjusting it for subcellular localizations; creating the Markov transition distribution matrix, assembling SCZ signatures from genome, epigenome, and transcriptome sources followed by creating the signal vector and adjust it for different tissues and cell types within them; creating tissue/cell-specific interaction networks, and signal diffusion across all of the dedicated networks to measure the disease signal intensities in unannotated proteins. Each dot on the human body scheme denoted the tissue being evaluated.

chromatin regions in neuronal cells.[30] The corresponding Ensembl IDs for all of these loci were obtained and the final signal matrix was created. Since MAPSD operates at the single-cell resolution, it needs to adjust the created initial signal vector *S* based on the tissues as well as their corresponding cell types to project the variations between the protein abundances among them (see Experimental Procedures). To illustrate elements of the vector *S*, suppose a gene to be DE and differentially methylated in SCZ compared with controls. Then, the initial signal intensity of this gene in *S* equals 2. Using the available protein abundance data in various tissues and cell types from the Human Protein Atlas, we adjusted the signal vector *S* for 131 combinations of tissues and cell types (Figure 2B, see Experimental Procedures). For instance, we have five regions in the brain, including cerebral cortex, cerebellum, caudate, hippocampus, and hypothalamus, as well as seven cell types, including neuronal cells, Purkinje cells, glial cells, endothelial cells, neutrophils, and cells in granular and molecular layers. Protein abundances vary across tissues and cell types. Therefore, it is required to overlay the knowledge on such expression patterns onto the signal vector *S*. The adjusted signal matrix is called S^* which shows the signal intensities of SCZ risk genes in all of the considered tissues and cell types. In fact, S^* reflects the functional consequences of genetic variants in distinctive tissues or cells, given that if the protein product corresponding to a genetic variant is lowly expressed in a specific tissue, then its functional impact will be lower compared with the tissues where its expression is higher. As a result, the number of candidate risk genes arising from propagation of signals through these proteins will be smaller. An important point to consider is to preserve the consistency between the omics data used to create the signal vector *S* and the context of the disease being studied. For example, in this study the data used to create the signal vector have been predominantly generated from the same brain region or appropriate surrogate tissues, otherwise this will result in spurious signals leading to false-negative predictions. In the next step, using the Markov operator matrix *M* and the created tissue/cell-specific signal intensity matrix S^*, MAPSD diffuses the available adjusted signal intensities onto the adjusted networks aimed at estimating the disease signal intensities of the unknown proteins (see Experimental Procedures). Upon termination of the algorithm, MAPSD outputs the signal intensities of all of the proteins in 131 different combinations of tissues and cell types, on which we conducted several tests. The MAPSD results are unbiased given that the adjusted network for signal diffusion is independently created from SCZ signal intensities and does not contain any prior information of the disease. Given that the PPI network is adjusted for subcellular localization of the nodes, the overall topology of the network shows a more realistic picture of subcellular molecular trafficking and protein interactions. The lower panel in Figure 1 represents a toy example of diffused signals as well as the original SCZ signal intensities in two different cell types. Given the abundance of proteins in each tissue and cell type, the overall diffusion patterns of SCZ signals varies in the two networks. The initial signal matrix does not include protein information. This information, including the localization in micro-domains and tissue-specific protein abundances, have been reflected in the model for adjusting the PPI network weights and create the affinity matrix as well as creating tissue-specific signal matrix, respectively.

Applying MAPSD on SCZ to Identify Disease Risk Genes

We created a large PPI network containing 232,801 edges and 16,185 nodes. As described above, considering five layers of omics evidences (gene expression, methylation, GWAS hits, rare and *de novo* mutation loci, and open chromatin regions), 3,915 genes were curated to be associated with SCZ with various degrees of signal intensities (Figure 3A). One gene (*DGKZ*) has a single intensity of 4 and six genes were found to have a signal intensity of 3, including *DNAJA4*, *TCF4*, *CHRNA2*, *CPNE8*, *GRIN2A*, and *ZNF536*. Notably, in a recent study[35] we

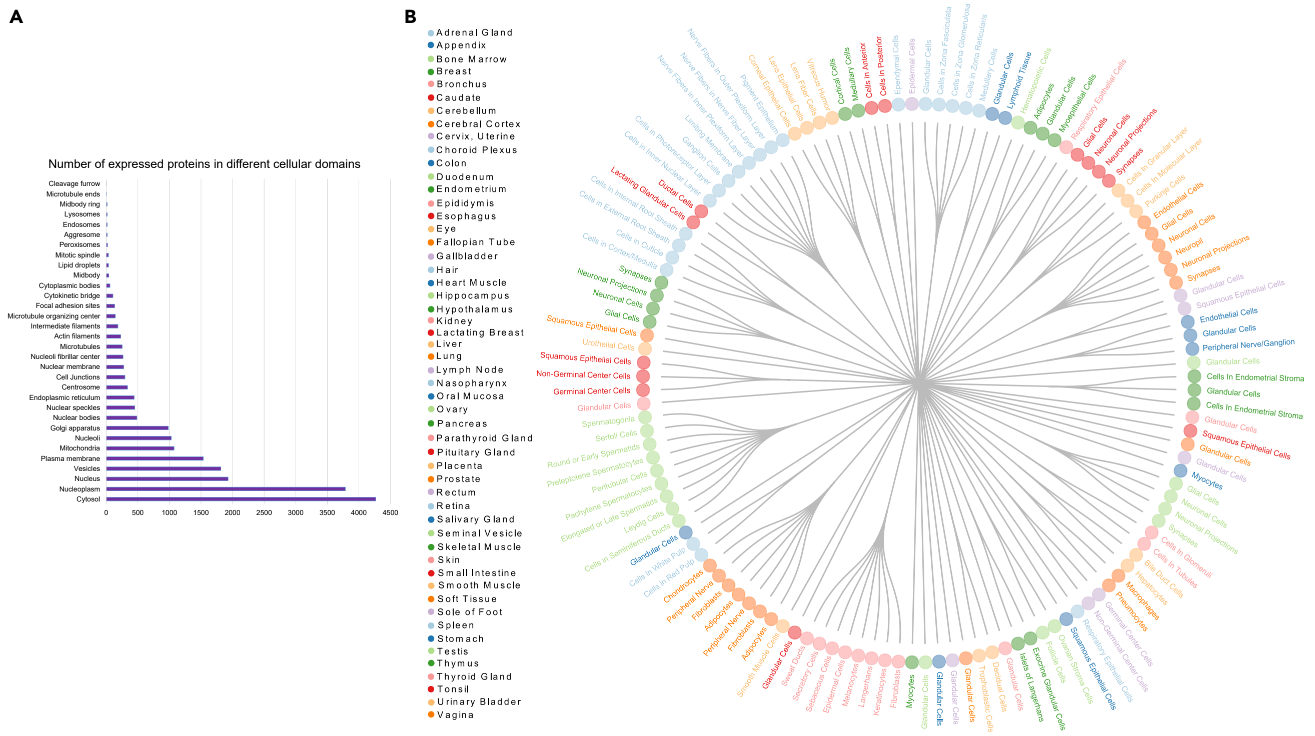

Figure 2. The List of Cell Types and Tissues Used in This Study

(A) The 131 combinations of cell types and tissues. Each color denotes a tissue and the forks for each color represent their corresponding cell types in this study.
(B) The list of subcellular domains in this study followed by the number of proteins being expressed in each subcellular domain.

had identified *TCF4* to act as a transcriptional master regulator in SCZ, based on expression network analysis of human dorsolateral prefrontal cortex. Upon initiating the diffusion process, MAPSD terminated the diffusion at the time step $t = 3$ (Figure 3C). A sharp decrease in Figure 3C indicates the tendency of the graph toward over-smoothness. Therefore, $t = 3$ is an appropriate cutoff point to prevent this phenomenon. After completion of the diffusion process, we sought to check how many of the SCZ risk genes show the highest signal intensity in all of the brain regions (Figure 3B). We can see that *DGKZ* as well as two other genes *CHRNA2* and *GRIN2A* with a signal intensity of 3 were preserved in the brain. MAPSD resulted in 704 genes (4.4% of the total, see Table S1) to have the highest SCZ risk signal uniquely in several brain regions, including cerebral cortex, cerebellum, hippocampus, and caudate. We checked this gene set to look for the SCZ risk genes (which were used as the input to the method) showing the highest risk signal intensity upon executing the MAPSD. We found that 190 genes have the highest signal intensities only in the brain (the total height of bars in Figure 3B). We checked the signal intensity of the remaining SCZ-associated genes (n = 3,725). We found 3,480 genes to have the highest signal intensity in the brain as well as at least one other tissue other than the brain, while 245 genes showed higher risk signals in other tissues other than brain.

MAPSD-Identified SCZ Risk Genes Are Enriched in Specific Subcellular Domains in Neuronal Cells

To evaluate the reliability of the MAPSD-identified candidate risk genes, we separated the 704 identified genes with the highest signal intensity in the brain into two groups: 190 known SCZ risk genes and 514 newly identified genes (Figures 4A and 4B). Using the protein abundances from the Human Protein Atlas, we checked in what specific brain regions and cell types the protein products of these genes are expressed. Of 190 known SCZ risk genes, 126 genes (66.3%) were highly expressed in neuronal cells in the cerebral cortex while in total, 138 genes (~72.3%) of the entire gene set were highly expressed in various cell types in the cerebral cortex. We next sought to evaluate the set of newly identified genes in the brain. We made a similar analysis on the 514 newly identified gene set by MAPSD. Among them, 360 genes (~70%) were highly expressed in neuronal cells in the cerebral cortex. In total, 396 genes were highly expressed only in the cerebral cortex which accounts for 77% of the total number of the newly identified gene set. Notably, these observations reveal an agreement between the enrichment patterns of both gene sets and suggests reliable cell specificity of the MAPSD approach. This finding is in agreement with the cell types suggested to be underlying SCZ pathogenesis.[36] In an important study, Skene et al.,[36] investigated the enrichment of SCZ common variants in adult brain temporal cortex and prefrontal cortex. Cell types being studied in these regions included: astrocytes, oligodendrocyte progenitor cells, oligodendrocytes, microglia, pyramidal neurons, and cortical interneurons. In both regions, pyramidal neurons and interneurons shared the highest degree of enrichment of GWAS loci compared with the other cell types. Our observations also show that the identified risk genes, at the protein level, are predominantly highly expressed in neuronal cells compared with other available cell types in this region.

CellPress
OPEN ACCESS

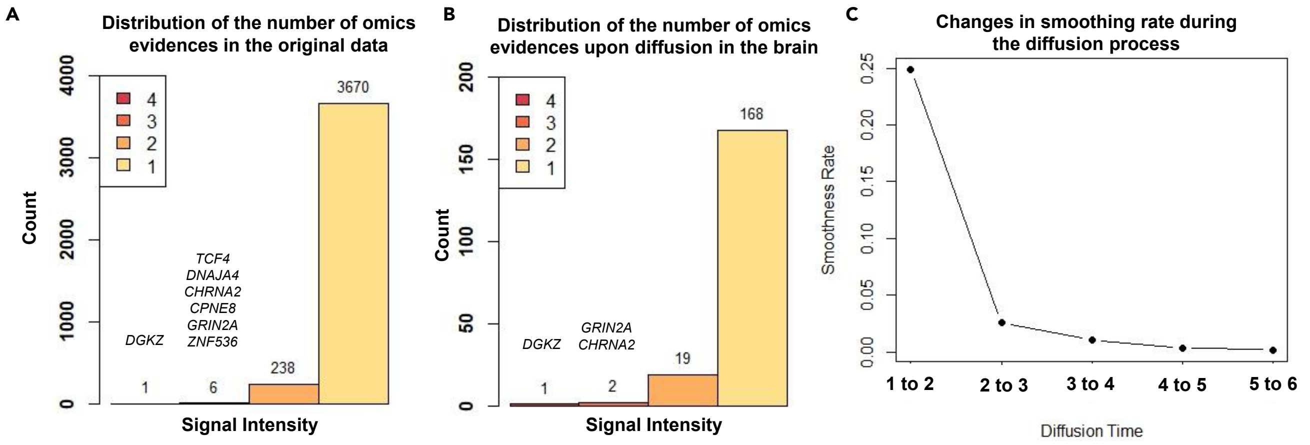

Figure 3. Distribution of SCZ Signal Intensities

(A) Distribution of initial signal intensities in the original signal vector.

(B) Distribution of initial signal intensities enriched in the brain after signal diffusion.

(C) Changes of smoothing rate during the diffusion time.

We also noted that endothelial cells share the lowest fraction of SCZ risk genes in our study. This is also the case in the findings of Skene et al., in which the enrichment of SCZ common variants in endothelial cells in prefrontal cortex is the lowest compared with the other cell types.

We were interested in finding the localization of SCZ risk genes in subcellular domains, using the subcellular localization domains obtained from Human Protein Atlas (Figure 2B). An immediate observation is significant enrichment of SCZ risk loci at protein level in various subcellular micro-domains of neuronal cells within the cerebral cortex (Figure 4C). Seventy-eight percent of the original SCZ risk genes found by MAPSD were enriched in neuronal cells in the cerebral cortex and across different subcellular domains. Among them, ~96% were enriched only in neuronal cells across different micro-domains. Further focusing on neuronal cells, we found that five micro-domains, including cytosol, nucleus, nucleoplasm, plasma membrane, and vesicles share ~70% of the entire SCZ-associated protein products in the cerebral cortex. Across the entire subcellular micro-domains, cerebellum harbors ~13% of the candidate SCZ risk genes, in which Purkinje cells shares the highest fraction of SCZ candidate risk genes at protein level.

We compared the enrichment patterns of the newly identified genes by MAPSD with the known SCZ risk genes based on their corresponding micro-domains. Similar to the SCZ risk genes, subcellular micro-domains in neuronal cells within the cerebral cortex share the largest fraction of the identified genes. We checked the newly identified gene set in the cerebral cortex. Considering all of the micro-domains, ~96% of the entire identified proteins are expressed predominantly in neuronal cells (Figure 4D). Within neuronal cells, five micro-domains share 72.5% of these proteins, including cytosol, nucleus, nucleoplasm, plasma membrane, and vesicles. This fraction is very similar to the localization of SCZ-associated protein products in neuronal cells within the cerebral cortex.

We compared the proportions of enrichment of SCZ genes and the identified genes based on their localizations within each cell in separate brain regions. In the cerebral cortex, considering all of the micro-domains and cell types, fractions of the both known SCZ risk genes and MAPSD newly identified genes were similar with no significant difference observed (chi-square p value = 0.79). We further compared the differences between the proportions of the major subcellular domains indicated above in neuronal cells within the cerebral cortex. Except vesicles (chi-square p value = 0.018), no significant difference was observed between their proportions: plasma membrane (chi-square p value = 0.9432), cytosol (chi-square p value = 0.114), nucleus (chi-square p value = 0.842), and nucleoplasm (chi-square p value = 0.191). These observations extend further support, regarding efficacy of MAPSD in modeling, a more realistic map of proteomic properties of SCZ at the cellular resolution.

MAPSD Recovers Potential Disease-Associated Susceptibility Protein Complexes

In addition to finding novel candidate risk genes, MAPSD can also reveal protein complexes that may be involved in disease pathogenesis. We tested MAPSD to show how it can facilitate recovering the SCZ risk signals in the brain. We ran MAPSD 100 times and each time randomly removed one SCZ risk gene with the highest signal intensity in the brain. MAPSD successfully recovered their signal intensities to bear the highest SCZ signal intensities in the brain. As an example, we illustrate the signal intensity of two SCZ risk genes (*DGKZ* and *ST8SIA2*) to show the highest signal intensity levels in the brain. *DGKZ* showed the highest signal intensity of 4. *DGKZ* is a well-studied SCZ risk gene demonstrated to be DE[2] and differentially methylated[28] as well as harboring GWAS hits[3,4] and *de novo* mutations.[26] MAPSD signal intensities for this gene (Figure 5A) are the highest in three regions, including neuronal cells in the cerebral cortex, Purkinje cells in the cerebellum, and neuronal cells in the caudate. *ST8SIA2* (Figure 5B) is known to be implicated in SCZ in various ways, such as its impacts on cerebral white matter diffusion properties in SCZ[37] as well as harboring multiple SCZ-associated SNPs.[3,38] After removing this gene from the initial signal vector, we ran MAPSD and observed that MAPSD

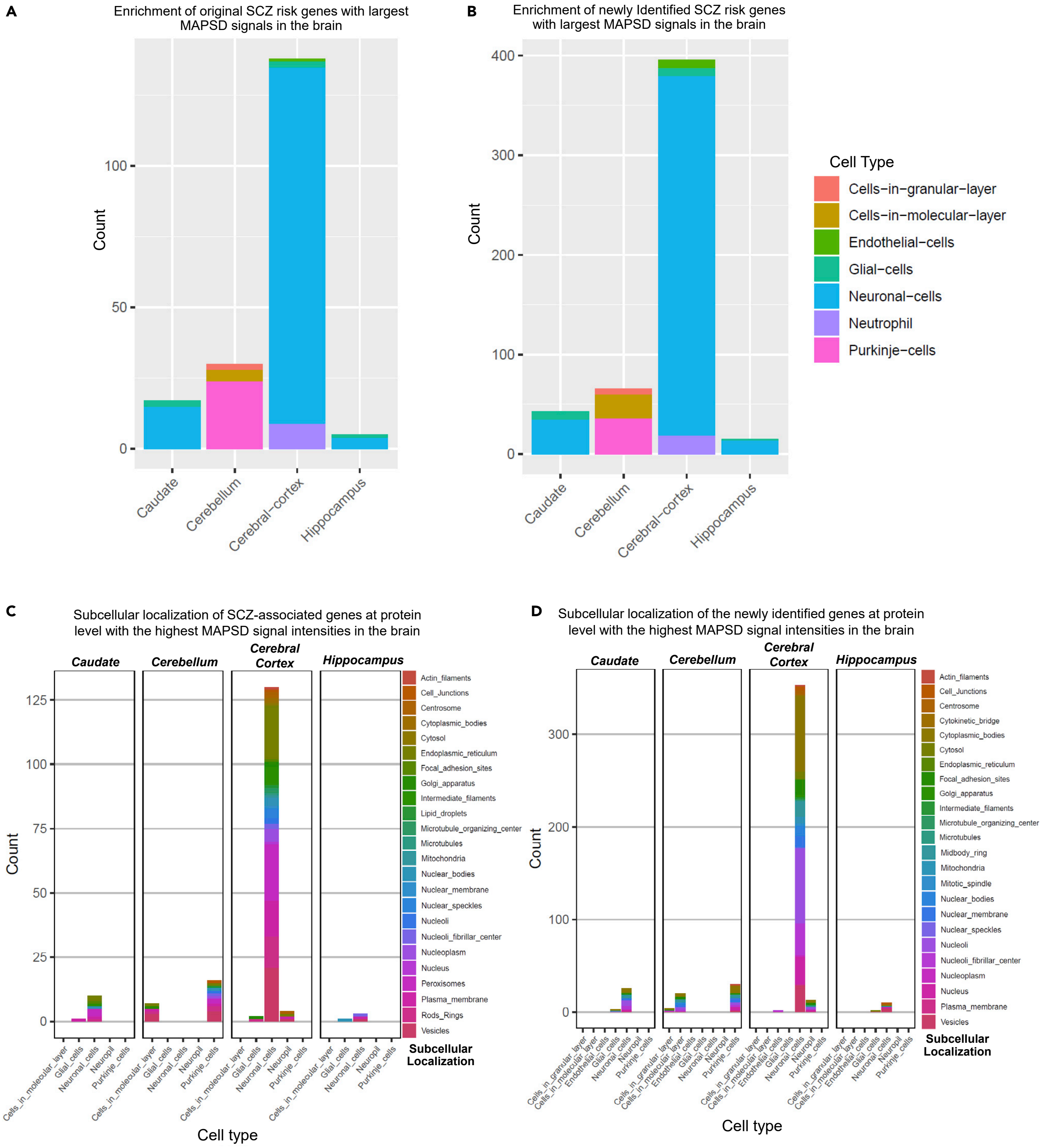

Figure 4. Expression Patterns of MAPSD Brain-Specific Genes at Cell Resolution and Subcellular Domains

(A) Frequency of MAPSD original SCZ risk genes at single-cell resolution to be highly expressed in four brain regions.

(B) Frequency of MAPSD newly identified SCZ risk genes at single-cell resolution to be highly expressed in four brain regions.

(C) Frequency of MAPSD original SCZ risk genes at protein level to be highly expressed in various subcellular domains in five cell types across four different brain regions.

(D) Frequency of MAPSD newly identified SCZ risk genes at protein level to be highly expressed in various subcellular domains in five cell types across four different brain regions.

CellPress
OPEN ACCESS

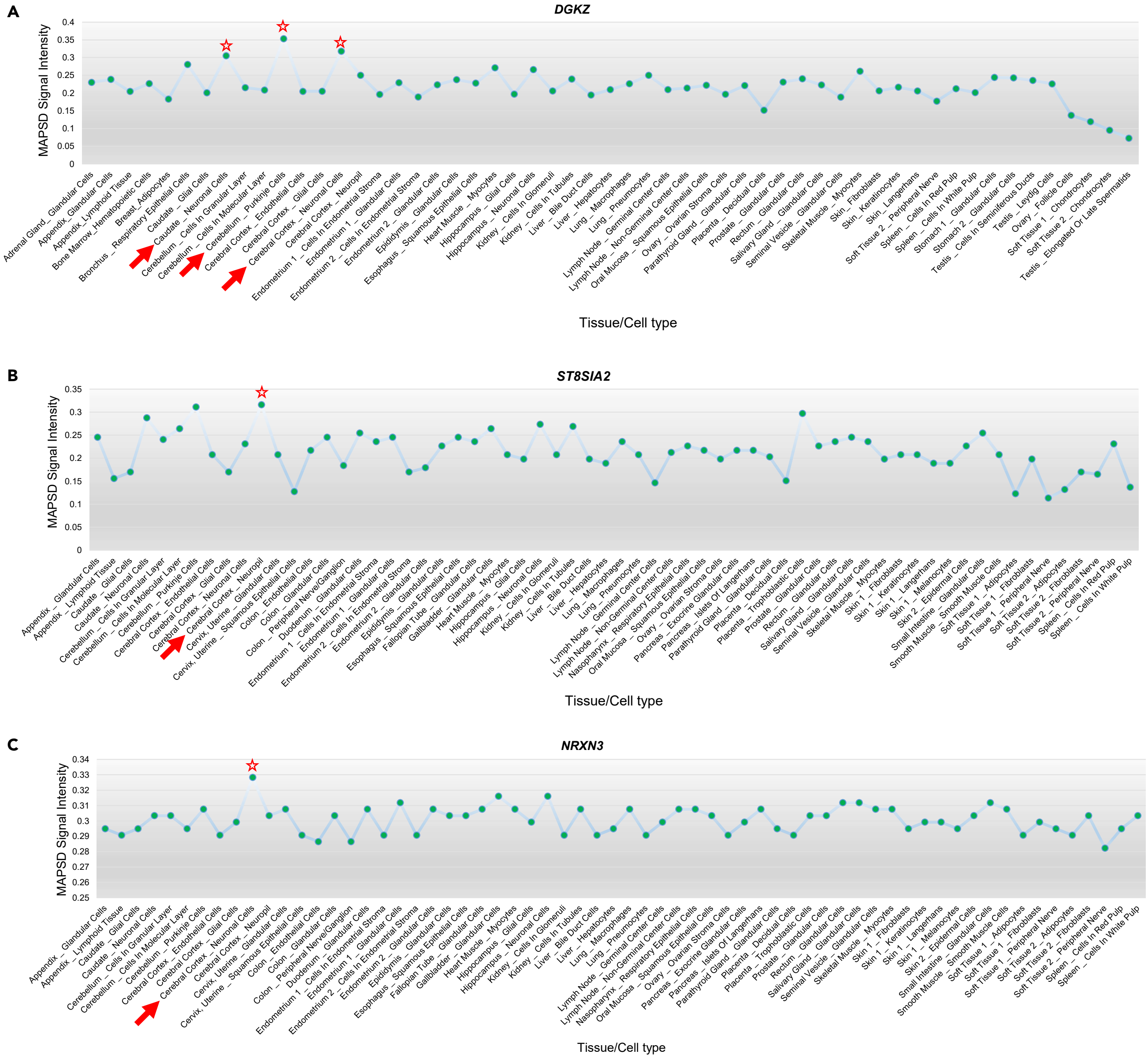

Figure 5. MAPSD Signal Intensities upon Diffusion in Three Genes

(A) MAPSD signal intensities of the SCZ risk gene DGKZ.

(B) MAPSD signal intensities of the SCZ risk gene ST8SIA2.

(C) MAPSD signal intensities of the gene DGKZ NRXN3 found to show the highest risk signals in the brain.

yields the highest SCZ signal intensities in the cerebral cortex and cerebellum. These experiments verify the robustness of MAPSD when the initial signal information for a disease is partially complete and that the method is capable to re-identify genuine SCZ risk loci given the topology of the adjusted PPI networks as well as proteome information incorporated into the model. Looking at the newly identified gene set by MAPSD, we found several genes to be implicated in other brain disorders. Considering that MAPSD can recover known SCZ-associated risk factors, we hypothesize that the newly identified genes may potentially be implicated in SCZ. On the other hand, we are already aware that many psychiatric disorders, such as SCZ, autism, and bipolar disorder share substantial genetic susceptibility.[39] Therefore, as a proof of concept, we picked some of the top MAPSD genes with the highest signal intensity and evaluated whether they have already been indicated in other brain diseases.

As a proof of concept, we picked *NRXN3*, which shows the highest signal intensity in neuronal cells in the cerebral cortex upon executing MAPSD (Figure 5C). The autism risk gene *NRXN3*[40,41] is a member of the Neuroxin gene family, which encodes neuronal adhesion proteins with critical roles in synapse development and function. Although restricted evidence, such as copy-number variation[42] and a polymorphism[43] on *NRXN3*

have been reported to be associated with SCZ in small population cohorts, its association to the disease has not been replicated[44] or widely recognized. We investigated the PPI network to look for the genes connected to *NRXN3*. *NRXN3* is directly connected to six genes, where the majority of them are significantly associated with diseases related to the central nervous system (CNS). These genes include *NLGN1*, *NLGN2*, *NLGN3*, *CASK*, *AFDN*, and *PAX4*. *NLGN1*, *NLGN2*, and *NLGN3* belong to the family of neuronal cell surface proteins, Neuroligin, and are involved in formation of CNS synapses.[45] They have been implicated in epilepsy,[46] autism spectrum disorders (ASDs),[47] and post-traumatic stress disorder.[48] Notably, MAPSD recapitulated these three genes in the brain where *NLGN1* and *NLGN2* were input to the model as SCZ risk genes, yet *NLGN3* was identified by MAPSD as a susceptibility disease risk gene. This finding is in concordance with the well-established observations that Neuroligin protein members act as ligands for Neuroxins, resulting in the connections between neurons and generation of synapses.[49] *CASK* and *AFDN* have also been implicated in CNS diseases such intellectual disabilities[50,51] and CNS leukemia,[52,53] respectively. Given that *AFDN* interacts with *NRXN3*,[54] we can conclude that MAPSD is capable of recovering high-risk loci in protein complexes and can infer converging disease risk modules in the human interactome.

Tissue and Developmental Stage-Specific Expression of MAPSD Risk Genes

To further gain evidence supporting their disease relevance, we analyzed the tissue-specific expression levels of the identified SCZ risk genes at mRNA level. For this analysis, we used gene expression levels on 53 different tissues from the Genotype-Tissue Expression (GTEx) project.[55] GTEx data contain mRNA levels across the entire transcriptome, which enables specifying to what extent a gene is expressed in distinct tissues. We divided the MAPSD risk genes into two groups, including the known SCZ risk genes with the highest signal intensities in the brain and newly identified genes with the highest signal intensity in the brain. We queried the GTEx data and observed that in both sets, the outputs of MAPSD are highly enriched in brain tissues (Figure 6A). In fact, frontal cortex showed remarkably higher enrichment scores, which is supported by the previous findings regarding its implications in SCZ.[2,56] The extent of enrichment in distinct brain regions was different. For instance, the frontal cortex and cerebral hemisphere represented a much stronger enrichment significance compared with other regions in the brain, while the amygdala and hippocampus, despite being significant, were less implicated in our analysis. In addition to the provided significance p values, we calculated the fold enrichment ratios (FER) for the top 5 significant brain regions for the set of identified genes, including frontal cortex (FER = 8.9), cortex (FER = 8.8), anterior cingulate cortex (FER = 21.7), nucleus accumbens (FER = 5.1), and cerebellar hemisphere (FER = 2.9). These observations suggest that integrating cell-specific genome and proteome knowledge in modeling the disease can lead to more sensitive and reliable identification of novel risk factors.

Because SCZ is likely a neurodevelopmental disorder, we next investigated if the brain-specific MAPSD genes are dysregulated during various developmental stages in human brain. We used the Atlas of the Developing Human Brain (BrainSpan)[57] on three brain regions, including the dorsolateral frontal cortex (DFC), cerebral cortex (CBC), and hippocampus (HIP). Next, we divided the data into two large categories of prenatal and postnatal stages, each with various time points. Prenatal stage includes 0–12 post-conception weeks (pcw), 13–24 pcw, and 25–36 pcw. Postnatal stages include 0–2, 3–8, 9–16, and >17 years. We averaged the expression levels of each MAPSD gene across different stages of pre- and postnatal stages and looked for DE genes (Figure 6B). Our observation indicates that almost half these genes were DE in postnatal stages versus the prenatal stages. The overall pattern of the number of DE genes in SCZ and MAPSD genes was almost similar. We were interested to specify what biological pathways are disrupted by the dysregulated genes during neurodevelopment in DFC, CBC, and HIP. We conducted pathway enrichment analysis (see Experimental Procedures) on these three gene sets. Although several pathways were nominally significant, none of them passed the false discovery rate (FDR) threshold of 0.05. On the other hand, checking the SCZ-associated genes that demonstrated the highest signal intensity while being DE during neurodevelopment led to finding multiple pathways that are statistically significant ($FDR < 0.05$). The majority of these pathways were shared by the three regions, such as glutamatergic synapse (DFC: $FDR = 2.3 \times 10^{-8}$, FER = 15.4; CBC: $FDR = 8.5 \times 10^{-9}$, FER = 13.8; HIP: $FDR = 2.8 \times 10^{-7}$, FER = 11.8), calcium signaling pathway (DFC: $FDR = 1.32 \times 10^{-7}$, FER = 10.4; CBC: $FDR = 8.5 \times 10^{-9}$, FER = 10; HIP: $FDR = 2.8 \times 10^{-7}$, FER = 8.7), circadian entertainment (DFC: $FDR = 7.5 \times 10^{-6}$, FER = 13.2; CBC: $FDR = 2.2 \times 10^{-7}$, FER = 13.6; HIP: $FDR = 4.4 \times 10^{-7}$, FER = 12.8), and cholinergic synapse (DFC: $FDR = 2.1 \times 10^{-5}$, FER = 11.3; CBC: $FDR = 7.3 \times 10^{-4}$, FER = 8.2; HIP: $FDR = 2 \times 10^{-4}$, FER = 8.8).

Some MAPSD Risk Genes Are Potential Drug Targets

We were interested in whether the MAPSD-identified SCZ risk genes act as targets of known drugs related to CNS. We used the list of US Food and Drug Administration (FDA)-approved drug targets by Santos et al.[58] comprising 4,631 drug-target connections as well as their mechanism of action. The data contained 881 unique protein targets in which the Ensemble IDs of 713 proteins were obtained. Among 514 newly identified MAPSD risk genes, we found 38 genes (Table S2) to be the targets of available FDA-approved drugs (FET p value = 2.68×10^{-4}). We found multiple calcium channel mRNAs to be of high-risk signal intensities, such as *CACNB1*, *CACNG2*, *CACNG3*, and *CACNG7*. These genes are known to be the targets of fragile X mental retardation protein, which cause fragile X syndrome and autistic symptoms.[59] These proteins were highly enriched in the brain, specifically in neuronal cells in the cerebral cortex (Figure 6C). We were interested in finding the genes that are already targets of drugs developed for CNS diseases. Twenty-one (56%) of the 38 genes were targets of drugs developed for CNS-related diseases (Figure S1). Some of these genes are well-documented risk loci in neurological diseases. For instance, *SCN1A*, a voltage-dependent sodium channel gene is known to be associated with epilepsy.[60,61] These genes are essential in generating action potentials in neurons and muscles. We found this gene to be the target of 16 drugs primarily developed to treat

CellPress
OPEN ACCESS

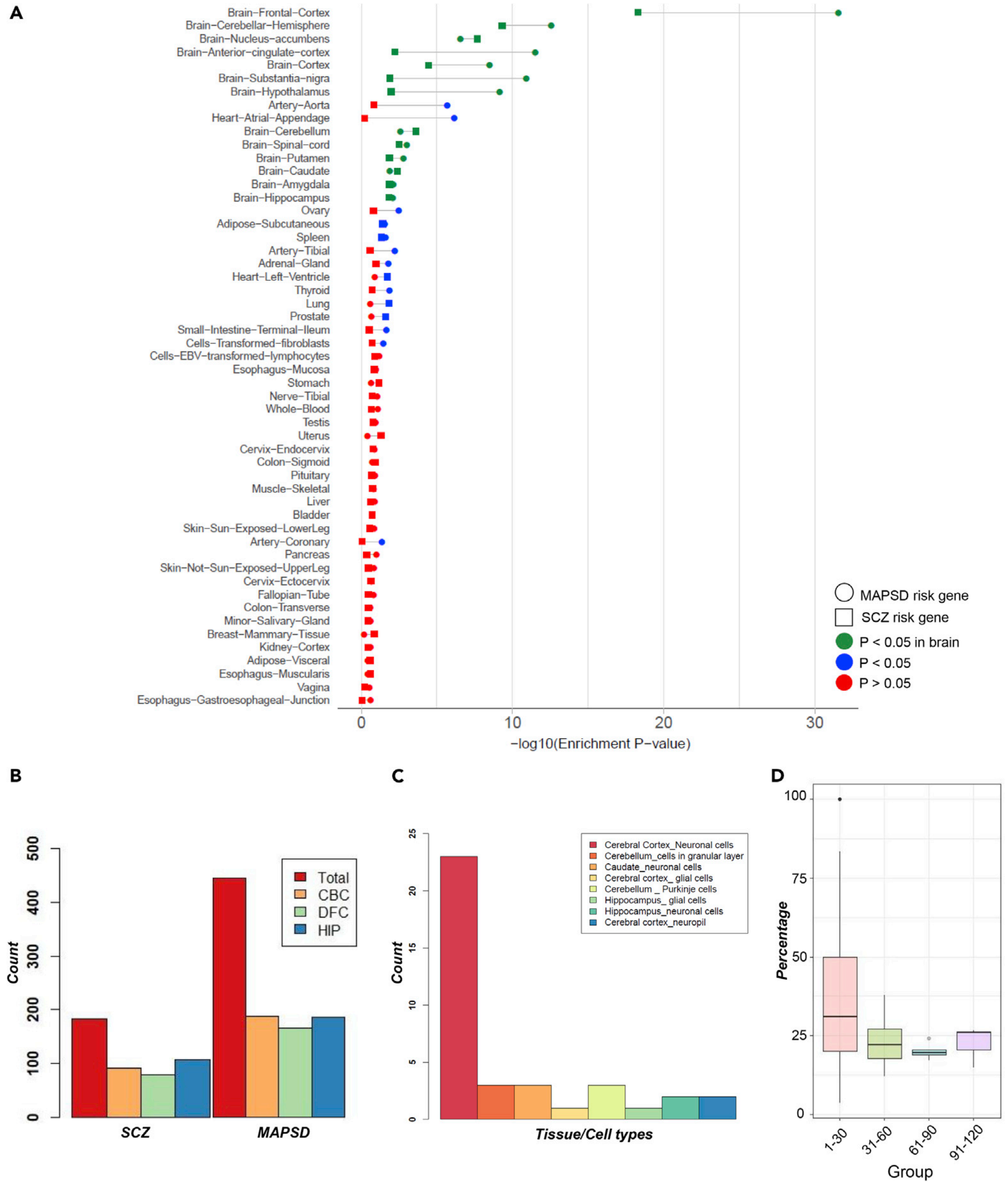

Figure 6. Tissue-Wise Enrichment Statistics for SCZ- and MAPSD-Identified Genes at Gene Expression Level

(A) –log10(p value) of SCZ and MAPSD risk genes with the highest signal intensity in brain tissues in GTEx consortium gene expression data.

(B) The number of differentially expressed SCZ and MAPSD risk genes in the cerebral cortex (CBC), dorsolateral frontal cortex (DFC), and hippocampus (HIP) between prenatal and postnatal developmental stages using BrainSpan data.

(C) Number of MAPSD risk genes to be the targets of FDA-approved drugs being enriched in specific cell types in certain brain regions.

(D) Percentage of SCZ-associated genes to be direct neighbors of the MAPSD-identified genes where each color represents MAPSD genes with a certain number of immediate connecting nodes in the PPI network.

epilepsy. We had found this gene to exhibit the highest signal intensity in neuronal cells in the cerebral cortex. Similarly, *SCN3A*, an epilepsy gene was picked up by MAPSD in neuronal cells in the cerebral cortex and hippocampus. These two genes have been widely studied in epilepsy as well as mental retardation and other neuropsychiatric disorders.[60] We recognize that these genes may have a different mode of action (gain of function versus loss of function) in different brain disorders, but our analysis demonstrated a proof of principle that MAPSD may facilitate drug repurposing efforts by integrating more fine-grained (tissue specific, cell-type specific, and subcellular localization specific) omics information on brain disorders.

Another highly connected gene within the created drug target network was *HRH1*. This gene was found to be the target of 51 drugs, of which 10 were developed for CNS diseases. This gene showed the highest MAPSD signal intensity in neuronal cells in the cerebral cortex despite not being used initially as an SCZ signature in MAPSD. A few studies have investigated its association with SCZ. For example, Nakai et al.[62] have shown the possible associations between *HRH1* and SCZ, despite

borderline evidence for an association in GWAS.[63] We found this gene to be connected to *ADRA1B* through two antipsychotic drugs chlorpromazine and trimipramine. Such interdependencies between the original SCZ risk genes supplied to MAPSD and the identified high signal genes further supports an orchestrated mechanism of the disease through interactions in convergent modules in the human interactome.

Among the identified genes to be drug targets, *CHRM1* and *CHRM2* were found to be targeted by over 30 drugs, 8 related to CNS. These genes are implicated in alcohol dependence,[64] major depression,[65] as well as possible involvements in SCZ.[66] In addition to the identified genes that might have been implicated in neuropsychiatric disorders, MAPSD revealed new candidates for treatment of SCZ. For instance, *SLC12A1*, a solute carrier transporter, was found with the highest signal intensity in the brain to be targeted by five drugs. This gene is essentially targeted to reduce edema caused by kidney or heart failure. However, granted the role of such membrane-bound proteins in transferring substrates within the cell, such as dopamine and serotonin,[67] they can be further studied for the treatment of SCZ.

DISCUSSION

In our view, the extreme polygenic nature of complex psychiatric disorders, such as SCZ, necessitates taking a more holistic view on the overall system of the diseases. One critical component of such a system is the proteome and its dynamics, given that proteins are in fact work horses of intra-cellular activities. Proteins reflect the genetic, epigenetic, and transcriptomic alterations that are caused by the disease. Yet, research on the proteome lags behind other omics data types, especially those generated on DNA and RNA levels,[14] due to technical limitations in data generation. Recent advances in proteome experimental paradigms has created new horizons to further use proteome knowledge in studying SCZ. Integrated analysis of omics data types at nucleic acid and amino acid levels makes it possible to accurately pinpoint SCZ drivers as well as accurate isolation of gene modules whose orchestrated interactions may confer susceptibility to the disease. Taking a multi-layer approach to SCZ, we introduced MAPSD, a proteogenomic signal diffusion method that accounts for subcellular localization of the proteins and intra-cellular trafficking in an integrated manner. Our study demonstrated the effectiveness of the MAPSD in recovering known SCZ risk genes and identifying novel candidate risk genes, and in identifying possible drug targets for drug-repurposing studies.

MAPSD has unique characteristics that are worth further discussion. MAPSD features modeling the protein localization in subcellular micro-domains as well as tissue-wise cell-specific distribution of protein abundances in the human body. Taking all this information into account, MAPSD creates a dedicated cell-specific PPI network for tens of distinct human tissues. This allowed us to create more realistic PPI networks that can lead to more accurate prediction of disease drivers. MAPSD jointly uses GWAS hits, DE genes, rare and *de novo* mutations, and chromatin accessibility data followed by diffusing this repertoire of information into each dedicated cell-specific PPI network to predict the signal intensities of novel candidate genes and their potential role in the disease onset and progression. The Markov affinity-based criterion borrowed from graph theory as well as the designed termination criterion ensures accurate transition of information across the network, while avoiding over-smoothing the signal intensities. Therefore, the highest amount of information will flow through the network while preventing the signals at each node are distinctive enough. MAPSD enables ranking the genes related to SCZ given their signal intensity levels in the brain.

An important strength of MAPSD is that the identified novel disease risk gene may not be immediate neighbors of known SCZ risk genes. For example, 217 genes out of 514 ($\sim$42%) identified risk genes by MAPSD are not directly connected to disease susceptibility loci. We checked the topology of the PPI network on the identified MAPSD risk genes, which were connected to at least one SCZ risk gene. Given the direct neighbors of MAPSD genes, we categorized them into four groups (Figure 6D) followed by counting the number of SCZ risk genes that are connected to each MAPSD risk gene within each group. Ninety-three percent of MAPSD genes have 1 to 30 direct neighbors among which the median percentage of SCZ risk genes is $\sim$30%. In other words, on average, 30% of the accumulated signals in MAPSD risk genes were transmitted directly from neighboring SCZ risk genes, while the remaining signal intensities are transmitted from distant genes. This is remarkable given that MAPSD can capture the signals from distant risk loci so that the convergence of small effect size loci can be observed and modeled. Another major property of MAPSD is its resilience against noise. Markov operators in graph signal processing act as a low-pass filter.[68] Therefore, in the case of introducing false signals, i.e., noise, to the MAPSD initial signal vector, these signals will automatically be filtered out during the signal diffusion. As a result, MAPSD is noise resistant. MAPSD was able to recover a significant fraction of known SCZ susceptibility genes from multi-omics studies. For example, in a recent study by Wang et al.,[5] multiple SNPs were reported to be associated with the disease. A significant overlap between MAPSD-identified genes and their reported loci was observed (FET p value = 2.1×10^{-4}, enrichment ratio = 3.2). Among them, 85% of the genes were enriched in neuronal cells in the cerebral cortex, 7.5% in Purkinje cells in the cerebellum, and 7.5% in neuronal cells in the caudate. This observation further supports the mechanism introduced in MAPSD to jointly model mutual interactions between omics data modalities for identification of novel risk genes and susceptibility risk modules in PPI networks.

Given that MAPSD takes an additive approach to combine signals from a variety of omics data types, we sought to explore if there were any correlations between these data types. We calculated the pairwise Matthews correlation among DE genes, *de novo* mutations, common variants, methylated loci, and open chromatin regions across the entire signature genes. Except for a mild correlation between DE and methylation signals (correlation coefficient = -0.43), we did not observe significant correlations between these data types. Incorporating the biophysical properties of proteins plays a critical role in precision predictions made by MAPSD. To evaluate the effects of removing the cell-specific PPI adjustment step in the performance of MAPSD, we ran MAPSD while disabling this stage followed by comparing the results with the original findings where the PPI network was adjusted for protein localization information. First, we looked for

the known SCZ risk factors showing the highest signal intensity in the brain. We found that 108 genes, compared with 190 genes when applying this stage, share the highest signal intensity in the brain demonstrating a 43% loss in reproducibility power of MAPSD. While all of the previous 190 genes were unique to the brain, we found 11 genes having unique signal intensities in tissues other than the brain. Regarding the prediction power of MAPSD, we came up with 450 genes to share the highest signal intensity in the brain compared with 514 predicted risk genes (12.5% decrease). These observations suggest a loss of power in reproducing a good portion of the predictions. Moreover, we evaluated the effect adjusting the signal vector using the cell-specific protein abundances. For this, we directly used the initial signal vector in the diffusion process in tandem with the adjusted PPI network. We found 69 SCZ risk genes, compared with 190 genes in the original experiment, to share the highest signal intensity in the brain. However, we found that 97 SCZ risk genes also show the highest signal intensity in other tissues, such as heart muscle, lung, and liver. Next, we checked the status of the 514 predicted hits in the original analysis. We found only 109 (21.2%) genes to be significantly enriched in the brain and 213 genes to be significantly enriched in other tissues. Collectively, it can be concluded that removing the effect of modeling the cell-specific protein abundances has a radical impact on the overall performance of MAPSD in distinguishing cell-specific hits. Moreover, we found, from above, that relaxing the PPI adjustment stage in MAPSD has serious negative impacts on the reproducibility power of the algorithm and diminished the overall reliability of the predictions.

To evaluate how MAPSD can be resilient to the networks being used, we conducted a secondary analysis using a second independent PPI network from the IMEx consortium[69] called the Interologous Interaction Database (I2D).[70] We observed 782 SCZ risk genes from the original signal vector of 3,915 risk factor to be present in the I2D PPI (~20% overlap). Among 190 SCZ risk factors which showed the largest signal intensity in the brain after running MAPSD, 45 genes existed in the I2D network, where 32 (71%) of them showed the highest signal intensity in the brain (Table S3). In our initial results, we had predicted 514 susceptible risk loci to share the highest signal intensity in the brain. Eighty-three of these predicted risk genes existed in the I2D network, where 55 (66%) genes yielded the highest signal intensity in the brain. We did not observe unique hits in the I2D PPI not being available in the analysis performed on our large curated PPI network. We had previously shown that SCZ risk genes *DGKZ*, *GRIN2A*, and *CHRNA2* (Figure 3) keep the highest signal intensity in the brain after the signal diffusion. Notably, two of them (*DGKZ* and *GRIN2A*) showed high signals in the brain after signal diffusion on the I2D network demonstrating a 67% overlap with the previous findings. Although the I2D PPI is significantly smaller (almost 18-fold) than the original PPI used previously, we were able to re-identify ~11% of the original predictions while only ~16% of the predictions existed in the I2D network. Therefore, our findings suggest that MAPSD is resilient to changing the networks being used. However, using a more detailed network will certainly lead to more robust predictions. In addition, conducting a randomized trial with 10 signal vectors each containing 3,915 signatures where none of which are SCZ risk loci led to an average of 473 genes with the highest signal intensity in the brain. We did not observe a significant overlap with the original SCZ findings ($p = 0.398$) suggesting the robustness of MAPSD.

There are some factors that may influence the overall quality of the prediction performance of MAPSD. First, the quality of the networks fed to the model. Since there are multiple compendia for PPIs, strict thresholds should be applied on the quality and reliability of pairwise interactions. This will ensure more accurate signal propagation through the network and will reveal more reliable outcomes. Second, more data types being fed to the model equates to more enriched signal matrices, which will in turn potentially lead to more concrete predictions regarding associations of the novel risk genes with the disease. If there is not enough evidence regarding each initial risk factor, then the Markov process will immediately converge while being over-smooth. Therefore, it would be difficult to interpret the findings, and the identified hits will likely be false negatives. Third, availability of high-resolution single-cell proteome data can increasingly improve the overall performance of MAPSD. Current data in the Human Protein Atlas are the major resource for profiling proteins across a wide range of tissues and cells. We acknowledge that the current data are not quite comprehensive, yet with the advent of technologies, generating more in-depth proteome data across more tissues has become possible. Therefore, we will be continuously updating MAPSD with more additional data. Given that a large number of the identified risk genes by MAPSD colocalize in various cell types in the cerebral cortex, we made sure that the results are not driven by the bias in the proteome data used. We used the Human Protein Atlas data and extracted the genes whose protein products are highly expressed in various cerebral cell types. Among 13,150 protein products, 2,894 (22.0%) proteins showed high expression in various cell types in the cerebral cortex. Therefore, the dataset used is not biased toward the cerebral cortex. MAPSD had predicted 514 novel risk genes among which 390 (~76%) are highly expressed in the cerebral cortex which is equivalent to an odds ratio of 3.45. Therefore, MAPSD findings are bias-free.

MAPSD takes advantage of high-dimensional omics data and is not tied to specific phenotypes. Therefore, it can effectively be applied to any complex disease, such as ASDs or autoimmune diseases, when necessary multi-omics datasets are available. MAPSD provides an ideal platform to leverage the outcomes of ongoing massive-scale projects, such as the PGC,[71] the largest consortium in psychiatry genetics, and the PsychENCODE project,[72] which is actively generating extensive epigenomic data on various psychiatric disorders. We envision MAPSD to be useful to the community to catalyze integrated evaluation of candidate genes for various neuropsychiatric and neurodevelopmental disorders at a systems level.

EXPERIMENTAL PROCEDURES

Resource Availability

Lead Contact

Kai Wang, PhD (email: wangk@email.chop.edu).

Materials Availability

This study did not generate any new unique reagents or materials.

Data and Code Availability

MAPSD scripts and data required for running the platform are available online at: https://github.com/adoostparast/MAPSD.

Description of the Data Used in the Study

Interaction networks used in this study were collected from three sources, including PICKLE 2.3,[33,34] the Human Reference Interactome,[32] and the Human Interactome Database.[31] Upon removing the duplicate interaction, the final network being used by MAPSD contained 232,801 interactions. The list of DE genes were obtained from the CommonMind Consortium.[2] GWAS hits on SCZ were downloaded from the CLOZUK consortium[4] and the Psychiatric Genomics Consortium.[3] Rare and *de novo* mutations were downloaded from denovo-db v.1.6.1.[26] DNA methylation data were downloaded from the works by Vitale et al.,[27] Aberg et al.,[28] and Alelu-Paz et al.[29] Open chromatin accessibility peaks were downloaded from the study by Bryois et al.[30] Protein abundances in all of the tissues and cell types as well as the subcellular localization of all of the proteins were obtained from the Human Protein Atlas project.[7,8] Tissue-specific gene expression levels were obtained from the GTEx project[55] consortium on 53 tissues.

Creating the Signal Vector

The initial signal matrix S, is an overlaid column vector which contains the cumulative levels of biological evidences, such as transcriptional signatures, methylation, GWAS. For each level of information for a specific gene, we add a point 1 if there was a significant hit, such as an FDR threshold of 0.05 on transcriptome signals and 5×10^{-8} for GWAS loci. To create S, first we introduce evidence matrix $E_{G \times L}$, where G denotes the total number of genes and L is the number of omics data layers (in this study, 5). Therefore

$$\begin{cases} E_{ij} = 1 & \text{if for gene } i \text{ there is evidence in layer } j \\ E_{ij} = 0 & \text{otherwise} \end{cases}$$

Next, using E, we can create S as follows: $S_i = \sum_{j=1}^{L} e_{ij}$. For example, if a gene i is DE and differentially methylated, then $S_i = 2$. We should make sure that the data being collected to create the signal vector have been generated from the same tissue or appropriate surrogate tissues to avoid generating spurious signals.

Adjusting the PPI Network Weights and Creating the Affinity Matrix

Subcellular localization data used in MAPSD were downloaded from the Human Protein Atlas project.[7,8] In total, 32 subcellular domains were available. To project this information onto the PPI network, first the affinity matrix A was created. A is an $n \times n$ binary matrix where $a_{ij} = 1$ if two proteins i and j are connected in the network, otherwise $a_{ij} = 0$. n denotes the total number of unique proteins in the PPI network. MAPSD scans the entire elements of A and checks its localization micro-domain. If two proteins i and j are connected in the network while co-localizing in the same micro-domain, then $a_{ij} = 1.5$. However, If two proteins i and j are connected in the network while not being co-localized in the same micro-domain, then $a_{ij} = 1$. Note that A is a symmetric matrix, i.e., $a_{ij} = a_{ji}$.

Creating the Markov Transition Matrix from Affinity Matrix

Upon adjusting the raw affinity matrix to contain the subcellular localization information, MAPSD obtains the Markov operator matrix (M). M is an $n \times n$ transition probability matrix whose element m_{ij} denoted the probability of single-step random walk from the node i to the node j. Leveraging random walk Laplacian in the graph theory,[73] M can be obtained as follows: $M = D^{-1}A$, where A denotes the adjusted affinity matrix above which consists subcellular localization information on all of the edges in the network and D represents the degree matrix. D is a diagonal matrix of the degree n, generated from A whose non-zero elements can be obtained as follows: $D_{ii} = \sum_{j=1}^{n} a_{ij}$. Therefore, each element of the main diagonal in D equals the row-wise summation of its corresponding protein in the affinity matrix A.

Creating Tissue/Cell-Specific Signal Matrix

To use the knowledge on the expression levels of each protein in each cell within each tissue, the Human Protein Atlas data were leveraged. In these data, expression levels are defined by four qualitative terms, including High, Medium, Low, and Not Detected. To use this in MAPSD, we converted them into a weight matrix $W_{G \times T}$, where G is the total number of proteins from the Human Protein Atlas and T is the total number of tissues and cell types. The total combinations of tissues and cell types in this study is 131. Therefore, the expression degree of protein i in the tissue/cell j is denoted by w_{ij} as follows:

$$w_{ij} = \begin{cases} \text{High} = 1 \\ \text{Medium} = 0.75 \\ \text{Low} = 0.5 \\ \text{Not detected} = 0.25 \end{cases}$$

Later, we converted the signal vector S to tissue/cell-specific signal matrix S^* by scalar multiplying the weight matrix W and the initial signal vector S as follows:

S^* is a $G \times T$ matrix where $S_j^* = W_j \odot S$, where matrix and $\odot$ denotes dot (scalar) product. Here, S_{ij}^* represents the disease signal intensity of the protein i in the tissue/cell j.

Signal Diffusion Process in MAPSD

MAPSD uses the Markov operator matrix M and tissue/cell-specific signal intensity matrix S^* to initiate the diffusion process. During the diffusion process, given the topology of the PPI network, for each combination of tissues and cell types, signal intensities of SCZ risk loci are propagated onto the network so the signal intensities of unknown proteins are estimated. The higher the signal intensity of a protein in the brain, the higher the likelihood of its association to SCZ. MAPSD is an iterative process where in each iteration signal intensities from disease risk genes are propagated through the network using the following equation: $S^t = M^t \times S^*$ where t denotes the diffusion time, i.e., the length of a random walk of size t from each node. A critical point to address during the diffusion process is choose of an appropriate diffusion time given that very large values of t leads to over-smoothness of the signal intensities. In other words, when the signals are over-smooth, then the signal intensities across all of the network will converge to a constant value leading to the loss of useful information. To avoid this situation, we have created a termination criterion called smoothness rate (R) as follows: $R = SSE/SST$, where SSE is the sum of square error and SST is the sum of square total and can be calculated as follows:$SSE = \sum_{i=1}^{G}\sum_{j=1}^{T} e_{ij}^2$, where e denotes a single element of the error matrix $E = M^{t+1}S^* - M^tS^*$. $SST = \sum_{i=1}^{G}\sum_{j=1}^{T} k_{ij}^2$, where k denotes a single element of the total matrix $K = M^{t+1}S^* + M^tS^*$. MAPSD terminates the diffusion process if $R \leq 0.05$. In other words, if the normalized difference of changes between signal intensities do not change at a certain threshold, then MAPSD stop the diffusion to avoid over-smoothing the signals of the protein across the network.

Pathway Enrichment Analysis

Pathway enrichment and gene ontology analysis were conducted using WebGestalt[74] v.2019. KEGG was used as the functional database the list of expressed genes were used as the background. The maximum and minimum number of genes for each category were set to 2,000 and 5, respectively, based on the default setting. Bonferroni-Hochberg multiple test adjustment was applied to the enrichment output. FDR significance threshold was set to 0.05.

SUPPLEMENTAL INFORMATION

Supplemental Information can be found online at https://doi.org/10.1016/j.patter.2020.100091.

ACKNOWLEDGMENTS

This study was supported by NIH grant MH108728 (to K.W.), MH106575 and MH116281 (to J.D.), Alavi-Dabiri Postdoctoral Fellowship Award (to A.D.T.), and CHOP Research Institute (to K.W.).

AUTHOR CONTRIBUTIONS

A.D.T. conceived the method, coded the algorithm, analyzed the results, and wrote the manuscript. J.D. provided guidance on the study design and the interpretation of results, and revised the manuscript. K.W. conceived the method, supervised the study, and edited the manuscript.

CellPress
OPEN ACCESS

DECLARATION OF INTERESTS

The authors declare no competing interests.

Received: May 6, 2020
Revised: July 1, 2020
Accepted: July 28, 2020
Published: September 2, 2020

REFERENCES

1. Wang, D., Liu, S., Warrell, J., Won, H., Shi, X., Navarro, F.C.P., Clarke, D., Gu, M., Emani, P., Yang, Y.T., et al. (2018). Comprehensive functional genomic resource and integrative model for the human brain. Science *362*, eaat8464.
2. Fromer, M., Roussos, P., Sieberts, S.K., Johnson, J.S., Kavanagh, D.H., Perumal, T.M., Ruderfer, D.M., Oh, E.C., Topol, A., Shah, H.R., et al. (2016). Gene expression elucidates functional impact of polygenic risk for schizophrenia. Nat. Neurosci. *19*, 1442–1453.
3. Schizophrenia Working Group of the Psychiatric Genomics Consortium (2014). Biological insights from 108 schizophrenia-associated genetic loci. Nature *511*, 421–427.
4. Pardinas, A.F., Holmans, P., Pocklington, A.J., Escott-Price, V., Ripke, S., Carrera, N., Legge, S.E., Bishop, S., Cameron, D., Hamshere, M.L., et al. (2018). Common schizophrenia alleles are enriched in mutation-intolerant genes and in regions under strong background selection. Nat. Genet. *50*, 381–389.
5. Wang, Q., Chen, R., Cheng, F., Wei, Q., Ji, Y., Yang, H., Zhong, X., Tao, R., Wen, Z., Sutcliffe, J.S., and Liu, C. (2019). A Bayesian framework that integrates multi-omics data and gene networks predicts risk genes from schizophrenia GWAS data. Nat. Neurosci. *22*, 691–699.
6. Harrison, P.J. (2015). Recent genetic findings in schizophrenia and their therapeutic relevance. J. Psychopharmacol. *29*, 85–96.
7. Uhlen, M., Fagerberg, L., Hallström, B.M., Lindskog, C., Oksvold, P., Mardinoglu, A., Sivertsson, Å., Kampf, C., Sjöstedt, E., Asplund, A., et al. (2015). Proteomics. Tissue-based map of the human proteome. Science *347*, 1260419.
8. Thul, P.J., Åkesson, L., Wiking, M., Mahdessian, D., Geladaki, A., Ait Blal, H., Alm, T., Asplund, A., Björk, L., Breckels, L.M., et al. (2017). A subcellular map of the human proteome. Science *356*, eaal3321.
9. Andersson, R., Gebhard, C., Miguel-Escalada, I., Hoof, I., Bornholdt, J., Boyd, M., Chen, Y., Zhao, X., Schmidl, C., Suzuki, T., et al. (2014). An atlas of active enhancers across human cell types and tissues. Nature *507*, 455–461.
10. Won, H., de La Torre-Ubieta, L., Stein, J.L., Parikshak, N.N., Huang, J., Opland, C.K., Gandal, M.J., Sutton, G.J., Hormozdiari, F., Lu, D., and Lee, C. (2016). Chromosome conformation elucidates regulatory relationships in developing human brain. Nature *538*, 523–527.
11. Mifsud, B., Tavares-Cadete, F., Young, A.N., Sugar, R., Schoenfelder, S., Ferreira, L., Wingett, S.W., Andrews, S., Grey, W., Ewels, P.A., and Herman, B. (2015). Mapping long-range promoter contacts in human cells with high-resolution capture Hi-C. Nat. Genet. *47*, 598–606.
12. Doostparast Torshizi, A., and Petzold, L.R. (2018). Graph-based semi-supervised learning with genomic data integration using condition-responsive genes applied to phenotype classification. J. Am. Med. Inform. Assoc. *25*, 99–108.
13. Jia, P., Chen, X., Xie, W., Kendler, K.S., and Zhao, Z. (2019). Mega-analysis of odds ratio: a convergent method for a deep understanding of the genetic evidence in schizophrenia. Schizophr Bull. *45*, 698–708.
14. Borgmann-Winter, K.E., Wang, K., Bandyopadhyay, S., Doostparast Torshizi, A., Blair, I.A., and Hahn, C.G. (2020). The proteome and its dynamics: a missing piece for integrative multi-omics in schizophrenia. Schizophrenia Res. *217*, 148–161.
15. Nagaraj, N., Wisniewski, J.R., Geiger, T., Cox, J., Kircher, M., Kelso, J., Pääbo, S., and Mann, M. (2011). Deep proteome and transcriptome mapping of a human cancer cell line. Mol. Syst. Biol. *7*, 548.
16. Schwanhausser, B., Busse, D., Li, N., Dittmar, G., Schuchhardt, J., Wolf, J., Chen, W., and Selbach, M. (2011). Global quantification of mammalian gene expression control. Nature *473*, 337–342.
17. Larance, M., and Lamond, A.I. (2015). Multidimensional proteomics for cell biology. Nat. Rev. Mol. Cell Biol. *16*, 269–280.
18. Arora, A., and Somasundaram, K. (2019). Targeted proteomics comes to the benchside and the bedside: is it ready for us? Bioessays *41*, e1800042.
19. Arrington, J.V., Hsu, C.C., Elder, S.G., and Andy Tao, W. (2017). Recent advances in phosphoproteomics and application to neurological diseases. Analyst *142*, 4373–4387.
20. Hada, V., Bagdi, A., Bihari, Z., Timári, S.B., Fizil, Á., and Szantay, C., Jr. (2018). Recent advancements, challenges, and practical considerations in the mass spectrometry-based analytics of protein biotherapeutics: a viewpoint from the biosimilar industry. J. Pharm. Biomed. Anal. *161*, 214–238.
21. Mardamshina, M., and Geiger, T. (2017). Next-generation proteomics and its application to clinical breast cancer research. Am. J. Pathol. *187*, 2175–2184.
22. Focking, M., Dicker, P., English, J.A., Schubert, K.O., Dunn, M.J., and Cotter, D.R. (2011). Common proteomic changes in the hippocampus in schizophrenia and bipolar disorder and particular evidence for involvement of cornu ammonis regions 2 and 3. Arch. Gen. Psychiatry *68*, 477–488.
23. Martins-De-Souza, D., Dias-Neto, E., Schmitt, A., Falkai, P., Gormanns, P., Maccarrone, G., Turck, C.W., and Gattaz, W.F. (2010). Proteome analysis of schizophrenia brain tissue. World J. Biol. Psychiatry *11*, 110–120.
24. Nesvaderani, M., Matsumoto, I., and Sivagnanasundaram, S. (2009). Anterior hippocampus in schizophrenia pathogenesis: molecular evidence from a proteome study. Aust. N. Z. J. Psychiatry *43*, 310–322.
25. Pennington, K., Beasley, C.L., Dicker, P., Fagan, A., English, J., Pariante, C.M., Wait, R., Dunn, M.J., and Cotter, D.R. (2008). Prominent synaptic and metabolic abnormalities revealed by proteomic analysis of the dorsolateral prefrontal cortex in schizophrenia and bipolar disorder. Mol. Psychiatry *13*, 1102–1117.
26. denovo-db, Seattle, WA (URL: denovo-db.gs.washington.edu) [accessed August 2019].
27. Vitale, A.M., Matigian, N.A., Cristino, A.S., Nones, K., Ravishankar, S., Bellette, B., Fan, Y., Wood, S.A., Wolvetang, E., and Mackay-Sim, A. (2017). DNA methylation in schizophrenia in different patient-derived cell types. NPJ Schizophr *3*, 6.
28. Aberg, K.A., McClay, J.L., Nerella, S., Clark, S., Kumar, G., Chen, W., Khachane, A.N., Xie, L., Hudson, A., Gao, G., and Harada, A. (2014). Methylome-wide association study of schizophrenia: identifying blood biomarker signatures of environmental insults. JAMA Psychiatry *71*, 255–264.
29. Alelu-Paz, R., Carmona, F.J., Sanchez-Mut, J.V., Cariaga-Martínez, A., González-Corpas, A., Ashour, N., Orea, M.J., Escanilla, A., Monje, A., Guerrero Márquez, C., et al. (2016). Epigenetics in schizophrenia: a pilot study of global DNA methylation in different brain regions associated with higher cognitive functions. Front. Psychol. *7*, 1496.
30. Bryois, J., Garrett, M.E., Song, L., Safi, A., Giusti-Rodriguez, P., Johnson, G.D., Shieh, A.W., Buil, A., Fullard, J.F., Roussos, P., and Sklar, P. (2018). Evaluation of chromatin accessibility in prefrontal cortex of individuals with schizophrenia. Nat. Commun. *9*, 3121.
31. Rolland, T., Taşan, M., Charloteaux, B., Pevzner, S.J., Zhong, Q., Sahni, N., Yi, S., Lemmens, I., Fontanillo, C., Mosca, R., et al. (2014). A proteome-scale map of the human interactome network. Cell *159*, 1212–1226.
32. Luck, K., Kim, D.K., Lambourne, L., Spirohn, K., Begg, B.E., Bian, W., et al. (2020). A reference map of the human binary protein interactome. Nature *580*, 402–408.

33. Gioutlakis, A., Klapa, M.I., and Moschonas, N.K. (2017). Pickle 2.0: a human protein-protein interaction meta-database employing data integration via genetic information ontology. PLoS One *12*, e0186039.

34. Klapa, M.I., Tsafou, K., Theodoridis, E., Tsakalidis, A., and Moschonas, N.K. (2013). Reconstruction of the experimentally supported human protein interactome: what can we learn? BMC Syst. Biol. *7*, 96.

35. Doostparast Torshizi, A., Armoskus, C., Zhang, H., Forrest, M.P., Zhang, S., Souaiaia, T., et al. (2019). Deconvolution of transcriptional networks identifies TCF4 as a master regulator in schizophrenia. Sci. Adv. *5*, eaau4139.

36. Skene, N.G., Bryois, J., Bakken, T.E., Breen, G., Crowley, J.J., Gaspar, H.A., Giusti-Rodriguez, P., Hodge, R.D., Miller, J.A., Muñoz-Manchado, A.B., et al. (2018). Genetic identification of brain cell types underlying schizophrenia. Nat. Genet. *50*, 825–833.

37. Fullerton, J.M., Klauser, P., Lenroot, R.K., Shaw, A.D., Overs, B., Heath, A., Cairns, M.J., Atkins, J., Scott, R., Schofield, P.R., and Weickert, C.S. (2018). Differential effect of disease-associated ST8SIA2 haplotype on cerebral white matter diffusion properties in schizophrenia and healthy controls. Transl. Psychiatry *8*, 21.

38. Arai, M., Yamada, K., Toyota, T., Obata, N., Haga, S., Yoshida, Y., Nakamura, K., Minabe, Y., Ujike, H., Sora, I., et al. (2006). Association between polymorphisms in the promoter region of the sialyltransferase 8B (SIAT8B) gene and schizophrenia. Biol. Psychiatry *59*, 652–659.

39. Gandal, M.J., Haney, J.R., Parikshak, N.N., Leppa, V., Ramaswami, G., Hartl, C., Schork, A.J., Appadurai, V., Buil, A., Werge, T.M., Liu, C., White, K.P., CommonMind Consortium; PsychENCODE Consortium; iPSYCH-BROAD Working Group, Horvath, S., and Geschwind, D.H. (2018). Shared molecular neuropathology across major psychiatric disorders parallels polygenic overlap. Science *359*, 693–697.

40. Vaags, A.K., Lionel, A.C., Sato, D., Goodenberger, M., Stein, Q.P., Curran, S., Ogilvie, C., Ahn, J.W., Drmic, I., Senman, L., and Chrysler, C. (2012). Rare deletions at the neurexin 3 locus in autism spectrum disorder. Am. J. Hum. Genet. *90*, 133–141.

41. Wang, J., Gong, J., Li, L., Chen, Y., Liu, L., Gu, H., Luo, X., Hou, F., Zhang, J., and Song, R. (2018). Neurexin gene family variants as risk factors for autism spectrum disorder. Autism Res. *11*, 37–43.

42. Ikeda, M., Aleksic, B., Kirov, G., Kinoshita, Y., Yamanouchi, Y., Kitajima, T., Kawashima, K., Okochi, T., Kishi, T., Zaharieva, I., et al. (2010). Copy number variation in schizophrenia in the Japanese population. Biol. Psychiatry *67*, 283–286.

43. Hu, X., Zhang, J., Jin, C., Mi, W., Wang, F., Ma, W., Ma, C., Yang, Y., Li, W., Zhang, H., et al. (2013). Association study of NRXN3 polymorphisms with schizophrenia and risperidone-induced bodyweight gain in Chinese Han population. Prog. Neuropsychopharmacol. Biol. Psychiatry *43*, 197–202.

44. Shavit, A., Gonzalez, J., Chavez, M., Rodriguez, M., Camarillo, C., Ramirez, M., Zavala, J., Contreras, J., Raventós, H., Flores, D., and Jerez, A. (2017). Association of NRXN3 deletion with schizophrenia and bipolar disorder. Biol. Psychiatry *81*, S206.

45. Davey, C., Tallafuss, A., and Washbourne, P. (2010). Differential expression of neuroligin genes in the nervous system of zebrafish. Dev. Dyn. *239*, 703–714.

46. Welberg, L. (2012). Synaptic plasticity: neuroligin 1 does the splits. Nat. Rev. Neurosci. *13*, 811.

47. Chen, J., Yu, S., Fu, Y., and Li, X. (2014). Synaptic proteins and receptors defects in autism spectrum disorders. Front. Cell Neurosci. *8*, 276.

48. Kilaru, V., Iyer, S.V., Almli, L.M., Stevens, J.S., Lori, A., Jovanovic, T., Ely, T.D., Bradley, B., Binder, E.B., Koen, N., et al. (2016). Genome-wide gene-based analysis suggests an association between Neuroligin 1 (NLGN1) and post-traumatic stress disorder. Transl. Psychiatry *6*, e820.

49. Scheiffele, P., Fan, J., Choih, J., Fetter, R., and Serafini, T. (2000). Neuroligin expressed in nonneuronal cells triggers presynaptic development in contacting axons. Cell *101*, 657–669.

50. Najm, J., Horn, D., Wimplinger, I., Golden, J.A., Chizhikov, V.V., Sudi, J., Christian, S.L., Ullmann, R., Kuechler, A., Haas, C.A., et al. (2008). Mutations of CASK cause an X-linked brain malformation phenotype with microcephaly and hypoplasia of the brainstem and cerebellum. Nat. Genet. *40*, 1065–1067.

51. Hayashi, S., Okamoto, N., Chinen, Y., Takanashi, J.I., Makita, Y., Hata, A., Imoto, I., and Inazawa, J. (2012). Novel intragenic duplications and mutations of CASK in patients with mental retardation and microcephaly with pontine and cerebellar hypoplasia (MICPCH). Hum. Genet. *131*, 99–110.

52. Xi, X.P., Zong, Y.J., Ji, Y.H., Wang, B., and Liu, H.S. (2018). Experiment research of focused ultrasound combined with drug and microbubble for treatment of central nervous system leukemia. Oncotarget *9*, 5424–5434.

53. Pinnix, C.C., Yahalom, J., Specht, L., and Dabaja, B.S. (2018). Radiation in central nervous system leukemia: guidelines from the international lymphoma radiation oncology group. Int. J. Radiat. Oncol. Biol. Phys. *102*, 53–58.

54. Hock, B., Böhme, B., Karn, T., Yamamoto, T., Kaibuchi, K., Holtrich, U., Holland, S., Pawson, T., Rübsamen-Waigmann, H., and Strebhardt, K. (1998). PDZ-domain-mediated interaction of the Eph-related receptor tyrosine kinase EphB3 and the ras-binding protein AF6 depends on the kinase activity of the receptor. Proc. Natl. Acad. Sci. U S A *95*, 9779–9784.

55. Ardlie, K.G., DeLuca, D.S., Segrè, A.V., Sullivan, T.J., Young, T.R., Gelfand, E.T., et al. (2015). The Genotype-Tissue Expression (GTEx) pilot analysis: multitissue gene regulation in humans. Science *348*, 648–660.

56. Weinberger, D.R. (1988). Schizophrenia and the frontal lobe. Trends Neurosci. *11*, 367–370.

57. Miller, J.A., Ding, S.L., Sunkin, S.M., Smith, K.A., Ng, L., Szafer, A., Ebbert, A., Riley, Z.L., Royall, J.J., Aiona, K., et al. (2014). Transcriptional landscape of the prenatal human brain. Nature *508*, 199–206.

58. Santos, R., Ursu, O., Gaulton, A., Bento, A.P., Donadi, R.S., Bologa, C.G., Karlsson, A., Al-Lazikani, B., Hersey, A., Oprea, T.I., et al. (2017). A comprehensive map of molecular drug targets. Nat. Rev. Drug Discov. *16*, 19–34.

59. Darnell, J.C., Van Driesche, S.J., Zhang, C., Hung, K.Y., Mele, A., Fraser, C.E., Stone, E.F., Chen, C., Fak, J.J., Chi, S.W., et al. (2011). FMRP stalls ribosomal translocation on mRNAs linked to synaptic function and autism. Cell *146*, 247–261.

60. Imbrici, P., Camerino, D.C., and Tricarico, D. (2013). Major channels involved in neuropsychiatric disorders and therapeutic perspectives. Front. Genet. *4*, 76.

61. de Lera Ruiz, M., and Kraus, R.L. (2015). Voltage-gated sodium channels: structure, function, pharmacology, and clinical indications. J. Med. Chem. *58*, 7093–7118.

62. Nakai, T., Kitamura, N., Hashimoto, T., Kajimoto, Y., Nishino, N., Mita, T., and Tanaka, C. (1991). Decreased histamine H1 receptors in the frontal cortex of brains from patients with chronic schizophrenia. Biol. Psychiatry *30*, 349–356.

63. Lee, S.T., Ryu, S., Kim, S.R., Kim, M.J., Kim, S., Kim, J.W., Lee, S.Y., and Hong, K.S. (2012). Association study of 27 annotated genes for clozapine pharmacogenetics: validation of preexisting studies and identification of a new candidate gene, ABCB1, for treatment response. J. Clin. Psychopharmacol. *32*, 441–448.

64. Luo, X., Kranzler, H.R., Zuo, L., Wang, S., Blumberg, H.P., and Gelernter, J. (2005). CHRM2 gene predisposes to alcohol dependence, drug dependence and affective disorders: results from an extended case-control structured association study. Hum. Mol. Genet. *14*, 2421–2434.

65. Cohen-Woods, S., Gaysina, D., Craddock, N., Farmer, A., Gray, J., Gunasinghe, C., Hoda, F., Jones, L., Knight, J., Korszun, A., and Owen, M.J. (2009). Depression Case Control (DeCC) Study fails to support involvement of the muscarinic acetylcholine receptor M2 (CHRM2) gene in recurrent major depressive disorder. Hum. Mol. Genet. *18*, 1504–1509.

CellPress
OPEN ACCESS

66. Dean, B., and Scarr, E. (2015). Possible involvement of muscarinic receptors in psychiatric disorders: a focus on schizophrenia and mood disorders. Curr. Mol. Med. *15*, 253–264.

67. Lin, L., Yee, S.W., Kim, R.B., and Giacomini, K.M. (2015). SLC transporters as therapeutic targets: emerging opportunities. Nat. Rev. Drug Discov. *14*, 543–560.

68. Ortega, A., Frossard, P., Kovačević, J., Moura, J.M.F., and Vandergheynst, P. (2018). Graph signal processing: overview, challenges, and applications. Proc. IEEE *106*, 808–828.

69. Orchard, S., Kerrien, S., Abbani, S., Aranda, B., Bhate, J., Bidwell, S., Bridge, A., Briganti, L., Brinkman, F.S., Cesareni, G., et al. (2012). Protein interaction data curation: the International Molecular Exchange (IMEx) consortium. Nat. Methods *9*, 345–350.

70. Brown, K.R., and Jurisica, I. (2007). Unequal evolutionary conservation of human protein interactions in interologous networks. Genome Biol. *8*, R95.

71. Sullivan, P.F., Agrawal, A., Bulik, C.M., Andreassen, O.A., Børglum, A.D., Breen, G., Cichon, S., Edenberg, H.J., Faraone, S.V., Gelernter, J., and Mathews, C.A. (2018). Psychiatric genomics: an update and an agenda. Am. J. Psychiatry *175*, 15–27.

72. Akbarian, S., Liu, C., Knowles, J.A., Vaccarino, F.M., Farnham, P.J., Crawford, G.E., et al. (2015). The PsychENCODE project. Nat. Neurosci. *18*, 1707–1712.

73. Newman, M.E.J. (2010). Networks : An Introduction (Oxford University Press).

74. Wang, J., Duncan, D., Shi, Z., and Zhang, B. (2013). WEB-based GEne SeT AnaLysis toolkit (WebGestalt): update 2013. Nucleic Acids Res. *41*, W77–W83.

PATTER, Volume *1*

Supplemental Information

Cell-Type-Specific Proteogenomic Signal Diffusion for Integrating Multi-Omics Data Predicts Novel Schizophrenia Risk Genes

Abolfazl Doostparast Torshizi, Jubao Duan, and Kai Wang

Supplementary Figures

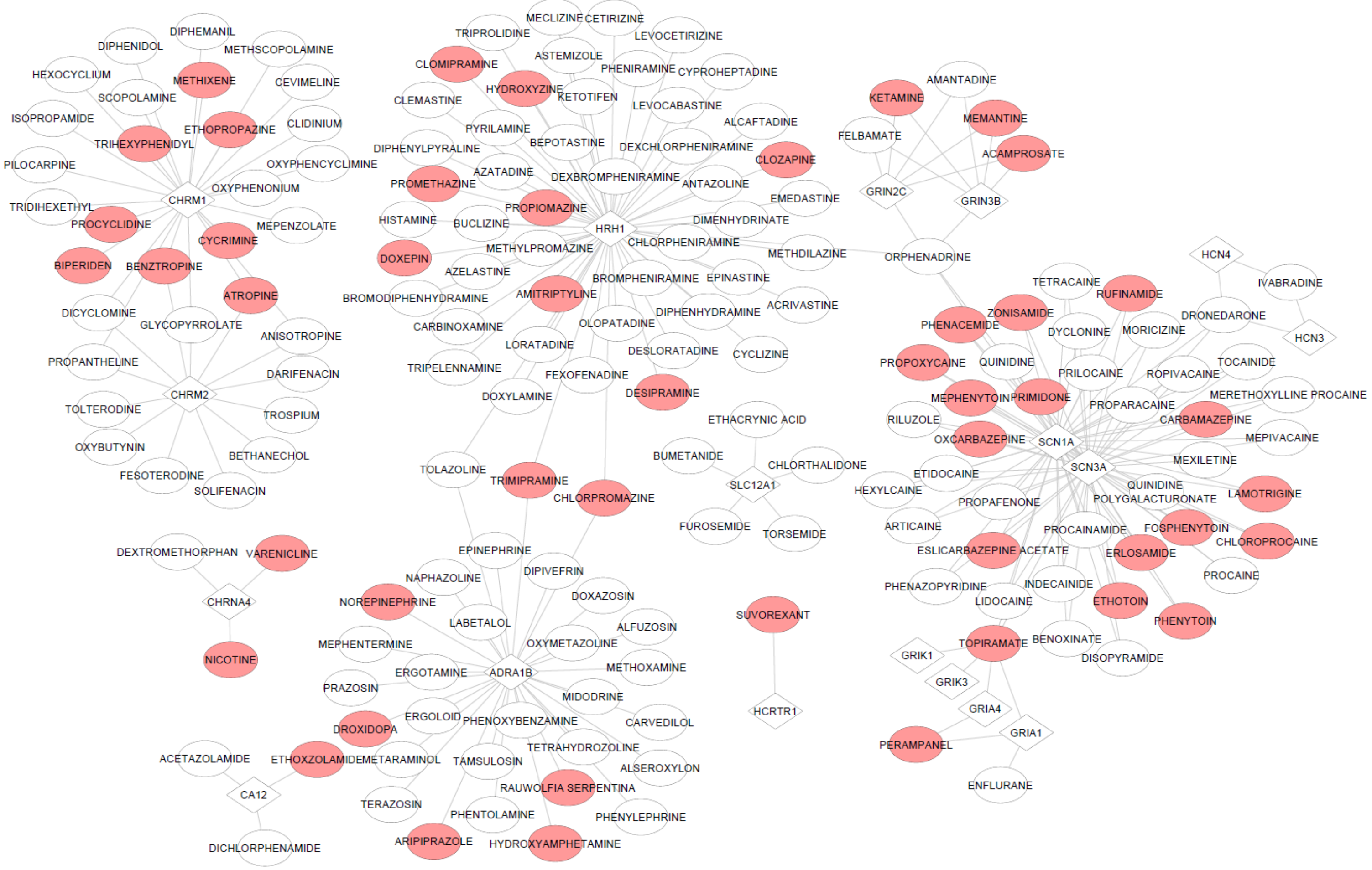

Supplementary Fig 1. Drug-target network representation of MAPSD risk genes and their corresponding FDA-approved drugs. Genes are denoted by diamonds and drugs are denoted by ovals. Red nodes represent the drugs developed for the diseases related to central nervous system.
